# An Improved CatBoost-Based Classification Model for Ecological Suitability of Blueberries

**DOI:** 10.3390/s23041811

**Published:** 2023-02-06

**Authors:** Wenfeng Chang, Xiao Wang, Jing Yang, Tao Qin

**Affiliations:** Department of Electrical Engineering, Guizhou University, Guiyang 550025, China

**Keywords:** blueberry, ecological suitability, CatBoost, Borderline-SMOTE, sparrow search algorithm

## Abstract

Selecting the best planting area for blueberries is an essential issue in agriculture. To better improve the effectiveness of blueberry cultivation, a machine learning-based classification model for blueberry ecological suitability was proposed for the first time and its validation was conducted by using multi-source environmental features data in this paper. The sparrow search algorithm (SSA) was adopted to optimize the CatBoost model and classify the ecological suitability of blueberries based on the selection of data features. Firstly, the Borderline-SMOTE algorithm was used to balance the number of positive and negative samples. The Variance Inflation Factor and information gain methods were applied to filter out the factors affecting the growth of blueberries. Subsequently, the processed data were fed into the CatBoost for training, and the parameters of the CatBoost were optimized to obtain the optimal model using SSA. Finally, the SSA-CatBoost model was adopted to classify the ecological suitability of blueberries and output the suitability types. Taking a study on a blueberry plantation in Majiang County, Guizhou Province, China as an example, the findings demonstrate that the AUC value of the SSA-CatBoost-based blueberry ecological suitability model is 0.921, which is 2.68% higher than that of the CatBoost (AUC = 0.897) and is significantly higher than Logistic Regression (AUC = 0.855), Support Vector Machine (AUC = 0.864), and Random Forest (AUC = 0.875). Furthermore, the ecological suitability of blueberries in Majiang County is mapped according to the classification results of different models. When comparing the actual blueberry cultivation situation in Majiang County, the classification results of the SSA-CatBoost model proposed in this paper matches best with the real blueberry cultivation situation in Majiang County, which is of a high reference value for the selection of blueberry cultivation sites.

## 1. Introduction

Blueberry belongs to the Vaccinium genus in the Rhododendron family and is also known as the “Queen of Fruits” because of its high nutritional value. Blueberry is famous in the market and the economic benefits brought by the blueberry industry appeal to the growing cultivation of blueberries in more and more countries [1,2,3]. In 2020, China’s blueberry planting area reached 66,400 hm^2^, surpassing the United States and ranking first in the world. Relying on its unique ecological and resource advantages, Majiang County in Guizhou Province, China, has planted 4700 hm^2^ of blueberries, of which 973.33 hm^2^ are organically certified, making it the most widely planted area with the best quality of fresh fruits in China and the development of the blueberry industry has contributed to the development of the local agricultural economy [4,5,6]. However, the quality and yield of blueberries are easily affected by ecological environments such as geography, meteorology, and soil. If it is planted in undesirable areas, hazards will occur frequently when growing, and when harvested, the yield of blueberry is low, with poor quality, low return on production investment and even resulting in losses [7]. Therefore, it is of practical significance to use modern electronic information technology to classify the ecological suitability of blueberries and reasonably select the planting areas of blueberries to further improve the yield and quality of blueberries and enhance its economic benefits.

A large number of researchers have studied the suitability of blueberries and the selection of their planting areas. Mo et al. [8] analyzed the influence of different meteorological indicators on blueberries in different phenological periods by selecting 14 various experimental observation sites for blueberry sampling and observation and using SPSS for correlation analysis, but the environmental factors were single, and the research analysis was not specific. Zhang et al. [9] established a multiple regression model for each meteorological and geographical factor by combining digital elevation data to classify the ecological suitability areas for blueberry cultivation in Guizhou Province, China, without combining soil data, which resulted in simple models and a low classification accuracy. Xiao et al. [10] took Highbush blueberry as the research object, identified the climate suitability characterization index, adopted the expert scoring method to establish the weight of each index, calculated the blueberry climate suitability indices, and divided the climate suitability areas for blueberry cultivation in Fujian Province, China based on GIS technology. This method only ascertained the influence of climate factors on blueberry, and the classification process had strong subjectivity. Qin et al. [11] utilized judgment matrix, linear weighting, and hierarchical cluster analysis to sketch potentially suitable areas for blueberry growth in Shaanxi Province, China, and the method also had the drawback of being highly subjective. Vera et al. [12] identified a territorial farm unit for setting up and managing blueberry crops on a farm in southern Chile, explored farm-level characteristics and classified ecological suitability and blueberry locations in the region based on their characteristics, but their applicability was weak. Cui et al. [13] studied blueberry suitability based on 783 blueberry geographical distribution records and 21 environmental variables and used a maximum entropy model and defined the potential distribution areas of blueberry globally and in China. However, due to the high dimensionality of the features, the training time of the model was long, the classification model was simple, and the classification accuracy was poor.

The ecological conditions suitable for the cultivation and growth of blueberry crops are multi-dimensional, specific, and comprehensively constrained. Existing research on the classification assessment of the environmental suitability of blueberry crops suffers from the following problems:

1. The classification model created a dataset with features containing only a single dimension and a sample of largely unbalanced data, so it fails to reflect multi-dimensionality.
2. Existing studies have used machine learning or artificial intelligence approaches to create a universal model for blueberry suitability classification, but the specificity of the model has led to more significant differences in the accuracy of blueberry suitability classification when the model is applied under different conditions.
3. Some factors in the development of blueberry crops can lead to changes in other elements under combined constraints. Current studies failed to consider this issue, resulting in simplistic model structures, long training time, and strong subjectivity.

With the development of artificial intelligence, machine learning technology has rapidly progressed and is applied in an increasing number of fields [14]. Many scholars have employed machine-learning methods in agriculture [15]. In smart agriculture, machine learning is mainly used to predict the yield of crops or identify crop diseases [16,17]. Still, there are still few applications in crop suitability classification [18,19], especially in the ecological suitability of blueberries. Therefore, this paper proposed a CatBoost-based classification method for the ecological suitability of blueberries. The core ideas of the method include the following:

1. It analyzed the applicability of different machine learning algorithms to classify blueberry ecological suitability. It surmounted the drawbacks of traditional machine learning models with simple structure and strong subjectivity. Additionally, the most suitable model for blueberry ecological suitability classification was identified.
2. Considering the constraints of comprehensive environmental factors on blueberry growth, the influence of some feature data on blueberry ecological suitability classification results were compared, which further confirms the applicability of the model.
3. The influence of different optimization algorithms (particle swarm optimization (PSO), whale optimization algorithm (WOA), and sparrow search algorithm (SSA)) on the parameters of classification models were explored to resolve the challenge of the long model training time and to improve the accuracy of classification results.

This study is the first to apply machine learning and multi-source environmental information fusion methods to crop planting site selection. Taking the blueberry growing area in Majiang County, Guizhou Province, China as an example, blueberry ecological suitability data were collected, and the Borderline-SMOTE algorithm was adopted to balance the number of positive and negative samples. Factors influencing blueberry growth were filtered out using variance inflation factors and information gain methods, with all features applied as inputs to each machine learning-based classification model. To further reduce the training time and improve the model’s performance, the paper employed various optimization algorithms to conduct the hyperparametric optimization of CatBoost. Based on the classification results of the other algorithms, the ArcMap 10.8 software was used to create a map of the ecological suitability of blueberries in Majiang County. The results were compared and validated with the actual blueberry planting situation in Majiang County.

## 2. Materials and Methods

### 2.1. Borderline-SMOTE Algorithms

**Figure 1:**
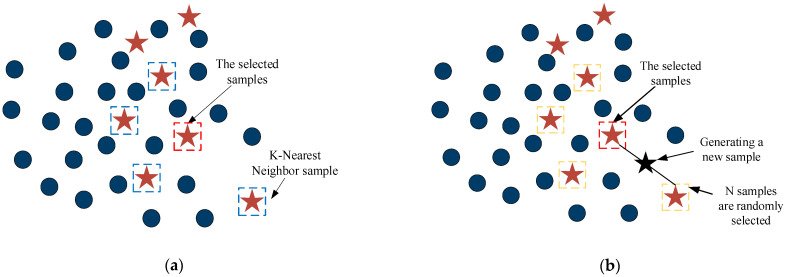
(**a**) Calculating the proximity of the selected sample K; (**b**) Synthesizing new samples.

In the general classification model dataset, there is a problem of imbalance in the number of sample labels. Models constructed from unbalanced data will have prediction results that are more biased towards brands with multi-category samples, which will lead to under-fitting or poor model results [20]. To balance the difference in the number of samples between categories and improve the efficiency of the classification model, the SMOTE algorithm was adopted to address the unbalanced data. The implementation process is shown in Figure 1.

Firstly, a sample $x_{i}$ was randomly selected from a minority class of samples. Secondly, N samples $\tilde{x}$ were randomly chosen from the K nearest neighbors of $x_{i}$ by sampling multiplicity N. Finally, new samples were synthesized in order randomly between $\tilde{x}$ and $x_{i}$, and the synthesis formula is as follows:

(1) $$x_{new}=x_{i}+rand\left(0,1\right)\times \left(\tilde{x}-x_{i}\right)$$

where $x_{new}$ is the “synthetic” sample, $\tilde{x}$ refers to the minority sample, $x_{i}$ denotes the chosen K-neighborhood sample, and rand(0,1) means producing any number between 0 and 1.

SMOTE treats all minority class samples equally and fails to consider the class information of the nearest neighbor samples, and there is sample overlap, leading to poor classification results. To solve the sample overlap problem brought about by the SMOTE algorithm sampling, Borderline-SMOTE [21,22] further improves this problem, and the elementary concept is as follows.

**Figure 2:**
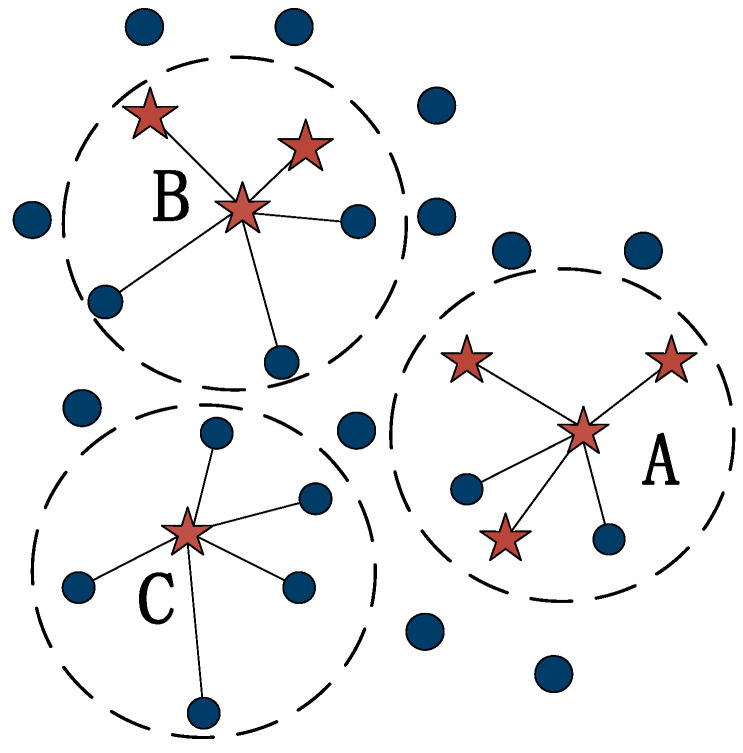
Classification rules for minority class samples.

(1) According to the Euclidean distance, the minority category samples are divided into three categories: *safe*, *danger*, and *noise*; *safe* represents the nearest neighbors more than the ordinary for the minority category; *danger* refers to the nearest neighbors more than half for the majority category samples; *noise* is the nearest neighbors all for the majority category samples, in turn, as marked by A, B, and C in Figure 2.
(2) A sampling ratio was set according to the imbalance rate, a sampling multiple was determined, and for each sample of a *danger* minority class, a number of samples were randomly drawn from its nearest neighbor samples.
(3) For each randomly selected nearest neighbor sample, a synthetic sample is obtained by the synthetic formula.

### 2.2. CatBoost Algorithm

CatBoost is derived from two words: “Categorical” and “Boosting”. It is an open-source machine learning algorithm developed by Yandex and widely used in R and python [23]. CatBoost is a GBDT (Gradient-Boosting Decision Tree) framework that is based on symmetric decision trees as primary learners with fewer parameters and supports class variables and high accuracy for the efficient and reasonable processing of class-type features. In addition, it also solves the problem of gradient bias and prediction shift, thus reducing the occurrence of overfitting [23,24]. In the decision tree, the label means will also be the criterion for node splitting, also known as greedy target variable statistics, and the formula is expressed as:

(2) $${\hat{x}}_{k}^{i}=\frac{\sum_{j=1}^{p-1}\left[x_{j,k}=x_{i,k}\right]\cdot Y_{i}}{\sum_{j=1}^{n}\left[x_{j,k}=x_{i,k}\right]}$$

**Figure 3:**
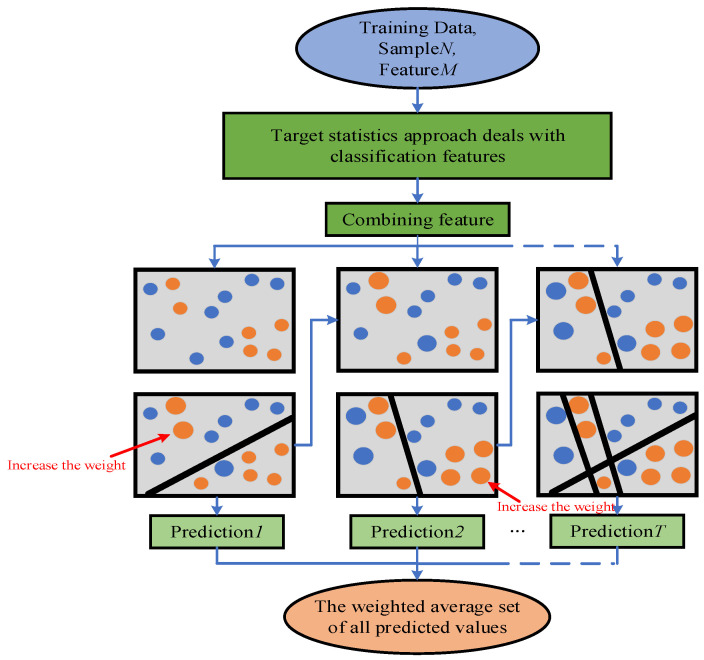
Flow chart of CatBoost algorithm.

This approach has the obvious drawback that features usually contain more information than labels. If the features are forced to be represented by the average of the labels, conditional bias can occur when the data structure and distribution of the training and test datasets do not match. A standard way of improving Greedy TS (Target-based Statistics) is to add a priori distribution terms to reduce the effect of noise and low-frequency category-type data on the data distribution, and the formula is expressed as follows:

(3) $${\hat{x}}_{k}^{i}=\frac{\sum_{j=1}^{p-1}\left[x_{\delta_{j,k}}=x_{\delta_{p,k}}\right]\cdot Y_{\delta_{j}}+a\cdot p}{\sum_{j=1}^{p-1}\left[x_{\delta_{j,k}}=x_{\delta_{p,k}}\right]+a}$$

where p is the added prior term and a typically refers to the weight coefficient greater than 0. For classification problems, the prior term is the prior probability of positive examples. At the same time, the algorithm will automatically combine categorical features into new features to further improve the expressiveness of the model. Given the above advantages of the CatBoost algorithm, the blueberry ecological suitability dataset itself is mostly characterized by categories, and the application of this algorithm can learn more information, and to the maximum extent, so as to further improve the model expression [25]. Figure 3 shows the Flow chart of the CatBoost algorithm.

### 2.3. Sparrow Search Algorithm

The sparrow search algorithm (SSA) [26,27] modeled the behavior of sparrow populations for predation and the warning of natural enemies, dividing the population into discoverers, followers, and scouts, with scouts subordinated to discoverers and followers. Discoverers have a high energy reserve in the population and are responsible for searching for food and guiding the movement of species in the population, usually making up 10–20% of the population, with the rest being followers. Followers forage in the direction guided by the discoverer, while constantly monitoring the discoverers and competing for food for predation rates. A random selection of 10–20% of individuals throughout the population act as scouts to warn of enemies.

The formula for updating the position of discoverers is as follows:

(4) $$X_{i,j}^{t+1}=\{\begin{array}{c} X_{i,j}^{t}\cdot exp\left(\frac{-i}{\alpha \cdot iter_{max}}\right)\;\;\;\;\;\;\;R_{2}<ST\\ X_{i,j}^{t}+Q\cdot L\;\;\;\;\;\;\;\;\;\;\;\;\;\;\;\;\;\;\;\;\;R_{2}\geq ST \end{array}$$

where $X_{i,j}^{t+1}$ denotes the position of the ith sparrow in the jth dimension at the current tth iteration. The $iter_{max}$ represents the maximum number of iterations, a random number within α∈(0,1), and $R_{2}\in \left[0,1\right],\;ST\in \left[0.5,1\right]$ represent the warning value and safety value, respectively. Q is the migration control coefficient, a random number obeying a standard normal distribution. L refers to a 1×d matrix with all matrix elements being 1. $R_{2}<ST$ means there are no natural enemies around and the discoverer conducts a global search; $R_{2}\geq ST$ means the warning value is reached, and the discoverer leads the population to escape from natural enemies.

The formula for updating the position of the followers can be seen as below:

(5) $$X_{i,j}^{t+1}=\{\begin{array}{c} Q\cdot exp\left(\frac{X_{worst}^{t}-X_{i,j}^{t}}{i^{2}}\right)\;\;\;\;\;\;\;\;\;\;\;\;\;\;\;\;\;\;\;\;\;\;\;\;i>\frac{N}{2}\\ X_{p}^{t+1}+\left|X_{i,j}^{t}-X_{p}^{t+1}\right|\cdot A^{+}\cdot L\;\;\;\;\;\;\;\;\;\;other \end{array}$$

where $X_{worst}$ denotes the current global worst position, $X_{p}^{t+1}$ refers to the optimal position of the *t*+1th iteration discoverer, A is a 1×d matrix with all matrix elements being 1 or −1, and $A^{+}=A^{T}{\left(AA^{T}\right)}^{-1}$. When $i>\frac{N}{2}$ indicates that this part of the followers is poorly positioned, in other words, low in adaptability and in hunger, they need to go to other places to forage for food.

The scout’s position update formula is as follows:

(6) $$X_{i,j}^{t+1}=\{\begin{array}{c} X_{best}^{t}+\beta \cdot \left|X_{i,j}^{t}-X_{best}^{t}\right|\;\;\;\;\;\;\;\;\;\;\;\;\;\;\;\;f_{i}>f_{g}\\ X_{i,j}^{t}+k\cdot \left(\frac{\left|X_{i,j}^{t}-X_{worst}^{t}\right|}{\left(f_{i}-f_{w}\right)+\varepsilon }\right)\cdot A^{+}\cdot L\;\;\;\;\;\;f_{i}=f_{g} \end{array}$$

where $X_{best}^{t}$ denotes the optimal global position, β is the step control coefficient and is a normally distributed random number with mean 0 and variance 1. k∈[−1,1] is a random number within *k* that denotes the orientation of the sparrow movement. ε is a tiny constant number that prevents the denominator from being zero. $f_{i}$ refers to the current adaptation value of the sparrow, $f_{g}$ and $f_{w}$ are the current global optimal and worst adaptation values, respectively. When $f_{i}>f_{g}$, it means that the sparrow is at the edge of the population and vulnerable to attack by natural predators; $f_{i}=f_{g}$ indicates that the sparrow in the center of the population senses the danger of being attacked by natural predators and needs to move closer to other sparrows.

## 3. Study Area and Data

### 3.1. Study Area

**Figure 4:**
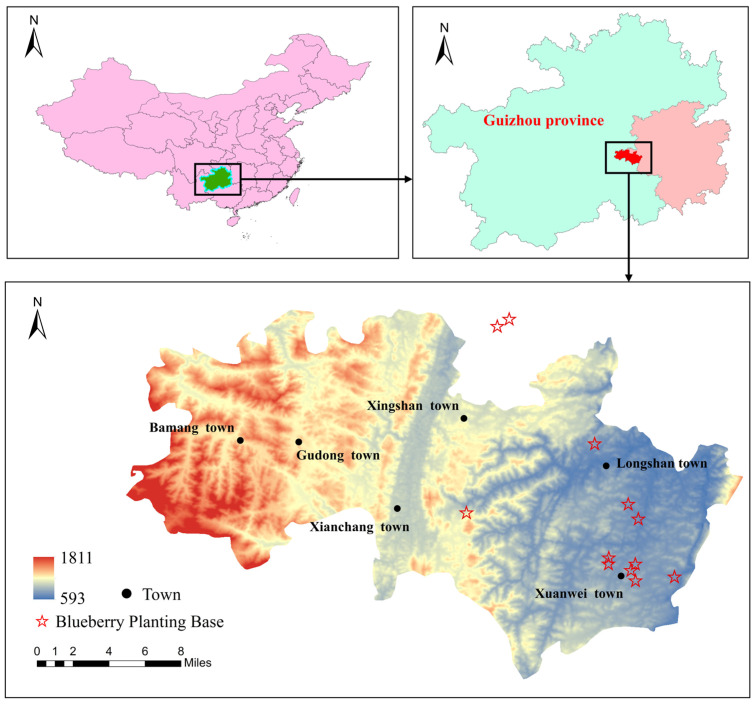
Overview map of the study area.

The study area is Majiang County (107°18′–107°53′ E, 26°17′–26°37′ N), located in the South East of the Guizhou Province, China. Majiang County has cultivated high-quality organic blueberry varieties based on its natural environment. There are 12 blueberry-growing sites in the county, each of which has a wealth of data on the ecological suitability of blueberries. An overview of the study area is shown in Figure 4.

### 3.2. Data Source

Geographic, meteorological, and soil factors are the most important features affecting blueberry suitability [7,11]. The data for this study consisted mainly of geographic, meteorological, and soil data. Geographic data were obtained from ASTER GDEM data of the Geospatial Data Cloud, including elevation, slope, slope aspect, and NDVI (Normalized Difference Vegetation Index). Meteorological data were sourced from the Guizhou Meteorological Bureau, including monthly average temperature, monthly precipitation, illumination intensity, and ≥10 °C cumulative temperature (March–September), in 2018. Soil data were collected from the Chinese Soil Database (Soil Science Database), including soil pH and soil organic carbon content. The primary data sources are shown in Table 1.

## 4. Modeling Process

### 4.1. Overall Framework

**Figure 5:**
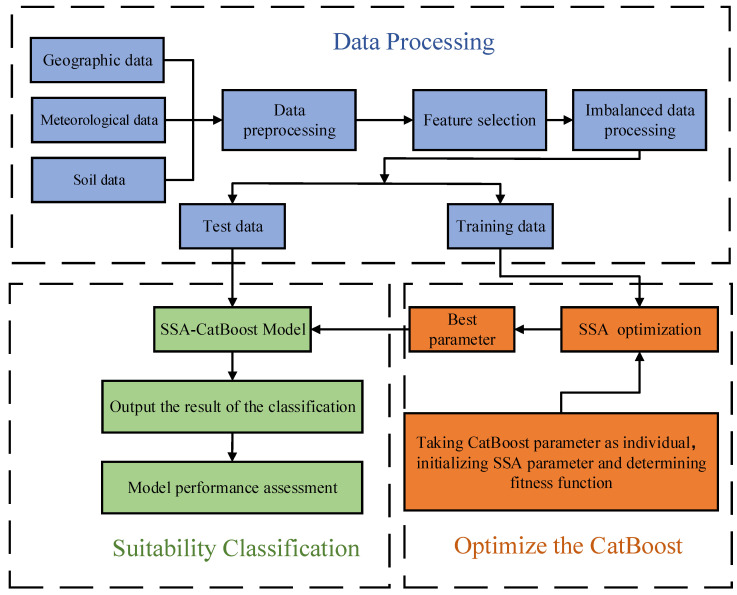
Overall frame diagram.

In this study, the CatBoost model was used to classify the ecological suitability of blueberries. It consists of three parts: data processing, optimize the CatBoost, and suitability classification. Firstly, the collected sample data was processed, including pre-processing, feature selection, and the processing of imbalanced data. Secondly, the parameters of the model were optimized using SSA to obtain the best parameters, so that the classification performance of the CatBoost model could be improved. Finally, the optimized model was used to classify the ecological suitability of blueberries and the model performance was evaluated. The overall frame diagram is shown in Figure 5.

### 4.2. Data Pre-Processing and Feature Selection

#### 4.2.1. Data Pre-Processing

The ecological suitability data of blueberries were obtained from 12 blueberry planting bases in LongBengShang, in Majiang County. Selected representative data are shown in Table 2. 

Among the ecological suitability feature data, there will be unreasonable data such as duplicate values, abnormal values, and missing values; at the same time, there will be problems such as the non-uniform size of different feature data, which will have an enormous impact on the prediction accuracy of the model. In this paper, the approaches for cleaning data are as follows.

1. Removal of duplicate data.
2. Filling of missing values, adopting the mean for continuous features and the plural for categorical features.
3. Data Bucketing, bucketing, and label encoding for continuous features according to the data distribution by quantile, and direct label coding of categorical features. The specific coding method can be seen in Table 3.

**Table 3:** Coding of suitability characteristics.

Number	Features	Coded Instructions
1	Elevation (m)	➀ <593; ➁ 593–816; ➂ 817–974; ➃ 975–1130; ➄ 1131–1328; ➅ 1329–1811; ➆ >1812
2	Slope (°)	➀ <10; ➁ 11–25; ➂ 25–35; ➃ 35–50; ➄ >50
3	Slope aspect	➀ Flat; ➁ North; ➂ North-east; ➃ East; ➄ Southeast; ➅ South; ➆ South-west; ➇ West; ➈ North-west
4	NDVI	➀ <3.5; ➁ 3.5–5.5; ➂ 5.5–9; ➃ >9
5	Monthly precipitation	➀ <50; ➁ 50–100; ➂ 100–170; ➃ 170–260; ➄ 260–300; ➅ >300
6	Monthly average temperature (°C)	➀ <0; ➁ 0–8; ➂ 8–15; ➃ 15–24; ➄ 24–30; ➅ >30
7	≥10 °C cumulative temperature (d·°C)	➀ <3000; ➁ 3000–4200; ➂ 4200–5500; ➃ >5500
8	Illumination Intensity (Lux)	➀ <1600; ➁ 1600–3200; ➂ 3200–4500; ➃ 4500–7000; ➄ 7000–10,000; ➅ >10,000
9	Soil pH	➀ <3.5; ➁ 3.5–4.5; ➂ 4.5–5.5; ➃ 5.5–7.1; ➄ >7.1
10	Soil organic carbon content (g/kg)	➀ <2; ➁ 2–4; ➂ 4–6; ➃ 6–8; ➄ >8

According to the coding rules of feature data in Table 3, the coding of each type of feature data in the sample data is shown in Table 4, where one denotes suitable ecological suitability of blueberry and zero indicates unsuitable.

#### 4.2.2. Feature Selection

The data were converted into features that could better indicate the potential problem of improving machine learning capabilities. In this paper, a filtered approach was adopted to screen blueberries for features, namely two statistical tests of the Variance Inflation Factor (VIF) and information gain (IG) [28].

VIF can identify and quantify multicollinearity problems. Multicollinearity occurs when there is a high degree of linear correlation between features of the input dataset, which may lead to incorrect modeling through fault analysis [29]. Based on the non-independence between elements, multicollinearity analysis assesses the reasonableness of the underlying assumptions used for the modeling rationale. VIF is calculated as follows:

(7) $$\mathrm{VIF}=\frac{1}{1-R^{2}}$$

where $R^{2}$ represents the correlation between features and the larger the $R^{2}$, the larger the VIF, meaning that there is a strong correlation between features. It is generally accepted that the closer the VIF is to one, the weaker the multicollinearity between features; VIF > five indicates strong multicollinearity.

IG is a measure of the information a feature can bring to the classified system [30], and the more information it brings, the more influential the feature is. The VIF and IG indices of ecological suitability features of blueberries are shown in Table 5 below.

In this study, the VIFs for the suitability features of blueberry were all less than five, and the IGs were all greater than zero, indicating weak multicollinearity among the features. Soil organic carbon content, elevation, Soil pH, ≥10 °C cumulative temperature, illumination intensity, and monthly precipitation contributed highly to the model, with each IG index being 0.271, 0.266, 0.198, 0.193, 0.136, and 0.128, respectively. It can be concluded that the selection of these ten-features data as inputs to the model training set allows for more adequate model training.

### 4.3. Imbalanced Data Processing

This paper collected sample data on the ecological suitability of blueberries from 12 planting bases in Majiang County, with a total of 918 cases. Among them, 632 cases were positive samples (samples with good suitability) and 286 cases were negative samples (samples with non-good suitability), which resulted in an imbalance in the proportion of samples. The classification results of the model constructed from imbalanced data would be more biased towards the labels of multi-category samples, which would lead to poor model fitting or poor results [31,32]. The random generation of negative samples was performed by the Borderline-SMOTE algorithm [33], and the distribution of the samples was balanced.

The experimental environment was Jupyter Notebook, and the package mainly includes python 3.8, Numpy, Pandas, SciKit-Learn, Imblearn, and other libraries. The sample of ecological suitability of the original blueberry was nine hundred eighteen cases with ten features. Bootstrap and Borderline-SMOTE methods were performed on the cleaned original data to obtain the required balanced dataset.

**Figure 6:**
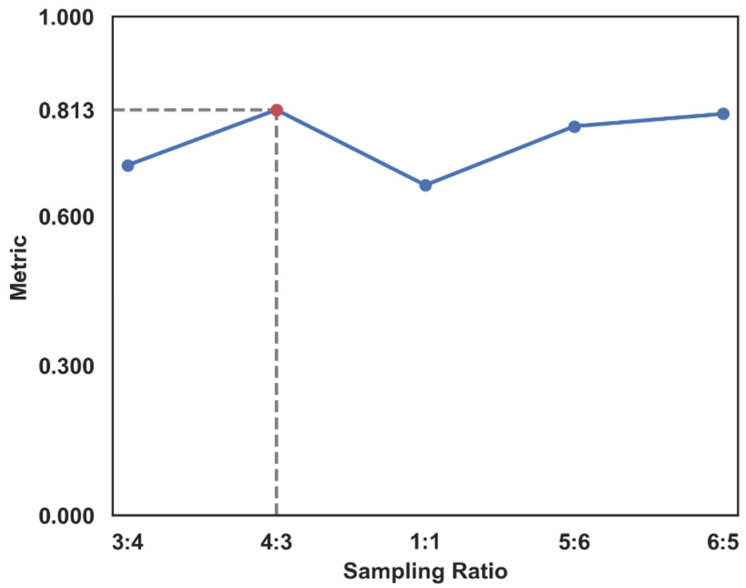
Sampling ratio selection.

To determine the sampling ratio used by the Borderline-SMOTE algorithm in the dataset [34], the Random Forest algorithm was used to test the sampled dataset. The Random Forest algorithm had better robustness and higher accuracy and is suitable as a model for testing. The AUC was selected as the evaluation index of the model, and the sampling ratios were chosen as 3:4, 1:1, 4:3, 5:6, and 6:5; the Random Forest model was adopted to construct the blueberry ecological suitability dataset and compared in turn, as shown in Figure 6.

Based on the effects achieved, the model was best assessed by oversampling the negative samples, keeping the ratio of samples roughly at 4:3, and the data set situation is shown in Table 6.

## 5. Results

### 5.1. Experimental Environment and Evaluation Metrics

The research experiment environment was Jupiter notebook, and the package mainly includes python 3.8, NumPy, pandas, SciKit-Learn, imblearn, and so on.

The classified problems must be evaluated in model evaluation by adopting different metrics to accomplish the iterative model training process. In this study, the precision, recall, comprehensive evaluation index (F1) [35], and area under-the-receiver operating characteristic curve (AUC) were used as evaluation metrics and calculated as follows [36]:

(8) $$\mathrm{Precision}=\frac{\mathrm{TP}}{\mathrm{TP}+\mathrm{FP}}$$

(9) $$\mathrm{Recall}=\frac{\mathrm{TP}}{\mathrm{TP}+\mathrm{FN}}$$

(10) $$F1-\mathrm{score}=\frac{2\times P\times R}{P+R}$$

AUC is the most critical metric for evaluating the model, which is the area under the receiver operating characteristic (ROC) curve. The prediction results of the model were sorted, and the samples were predicted as positive cases in order, and the True Positive Rate (TPR) and False Positive Rate (FPR) were calculated each time as the horizontal and vertical axes, respectively, and the formula is defined as follows:

(11) $$\mathrm{TPR}=\frac{\mathrm{TP}}{\mathrm{TP}+\mathrm{FN}}$$

(12) $$\mathrm{FPR}=\frac{\mathrm{FP}}{\mathrm{TP}+\mathrm{FP}}$$

The value of AUC is a vital evaluation index of the model, which is generally between 0.5 and 1, with larger values implying better generalization ability and classification performance [37].

### 5.2. Model Comparison

For the processed data, the 918 samples data were divided into training and testing sets in the ratio of 8:2, i.e., 80% of the data were used for the training of the model and 20% for the testing of the model. 

**Figure 7:**
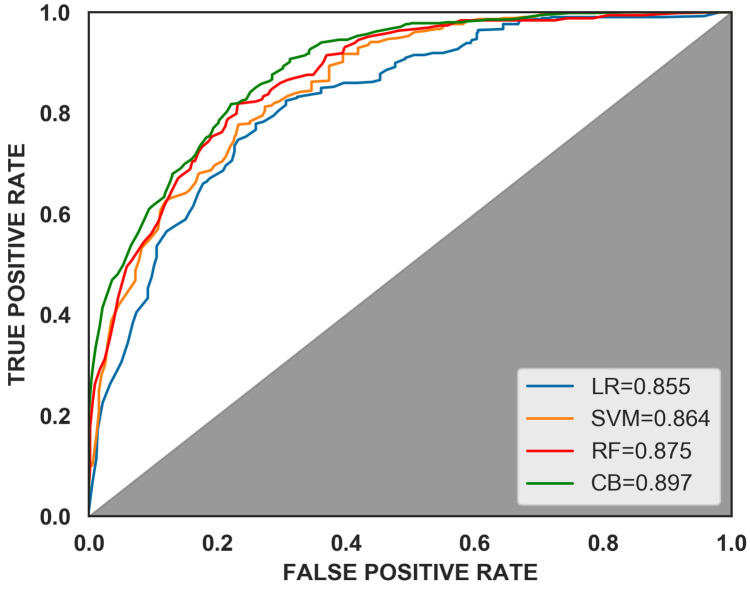
Curve comparison of ROC.

**Figure 8:**
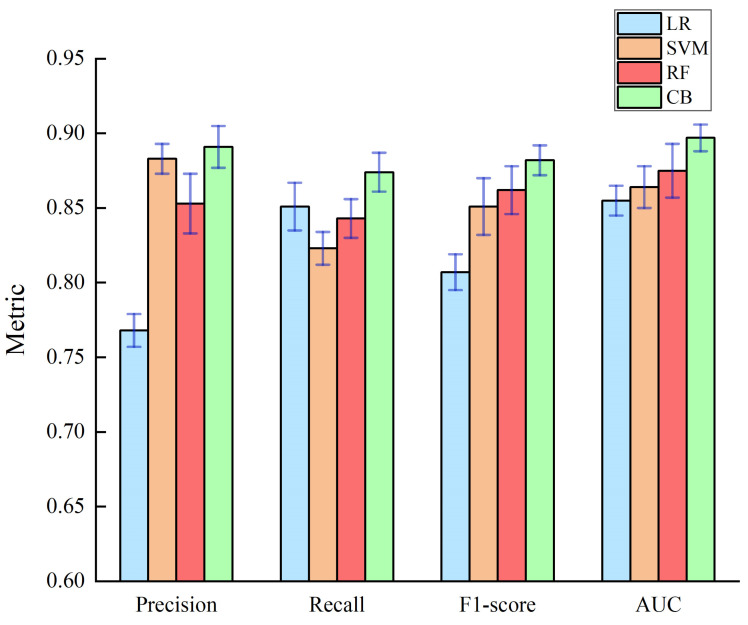
Standard deviations of different models in each evaluation index.

Four different classification models were built using Logistic Regression (LR), Support Vector Machine (SVM), Random Forest (RF) [38,39,40], and CatBoost; the models were parameterized using empirical settings, as shown in Table 7. To ensure the reliability of the experimental results and to avoid the chance of single experimental results, several experiments were conducted on different models. The comprehensive comparison of each model evaluation index is shown in Table 8, Figure 7 and Figure 8. The comparison of the training time of the models is shown in Table 9.

As shown by Figure 8 and Table 8, the proposed model scored the highest AUC value of 0.897, which was 4.91%, 3.82%, and 2.51% higher than LR (0.855), SVM (0.864), and RF (0.875), respectively. Besides, the CatBoost model also performed well in terms of the precision, recall, and F1-score. In terms of precision, the CatBoost model outperform the LR, SVM, and RF models by 16.01%, 0.91%, and 4.45%, respectively. Additionally, its recall was 2.70%, 6.20%, and 3.68% higher than that of the LR, SVM, and RF models, respectively. In terms of F1, the CatBoost model beats the LR, SVM, and RF models by 9.30%, 3.64%, and 2.32%, respectively. Therefore, the CatBoost model has the best classification performance.

As shown in Table 9, the training time of the LR and RF models was shorter, and the training time of the CatBoost model was 21 s, which was longer than that of the LR and RF models, while the training time of the SVM model was the longest. After analysis, the reasons for the above observations are as follows. The LR and RF models have lower complexity, so their training time is shorter; the SVM model has a higher kernel function complexity, so its training time is the longest; the CatBoost can learn the category features better due to prior classification of the category-based features of the dataset, and requires less training time, so the speed of classification prediction is faster.

### 5.3. Model Optimization

CatBoost was trained via the gradient boosting method. In the corresponding iteration, the basis for generating a new learner is the regularized objective function, too large or too small a regularization parameter L2_leaf_reg can lead to the over- or under-fitting of the model. For the learning rate, a small value will consequently cause the gradient to fall too slowly, while too large a value may miss the optimal value and generate oscillations. For the iterations, too small a parameter can lead to underfitting, resulting in inadequate model resolution, while too large a parameter can lead to overfitting, resulting in a decrease in the generalization ability of the model. In addition, the choice of depth is also important, as a wrong choice can affect the learning ability and classification capability of the model [41]. Therefore, in this study, an optimization algorithm was chosen to optimize the above four parameters to improve the performance of the classification model.

**Figure 9:**
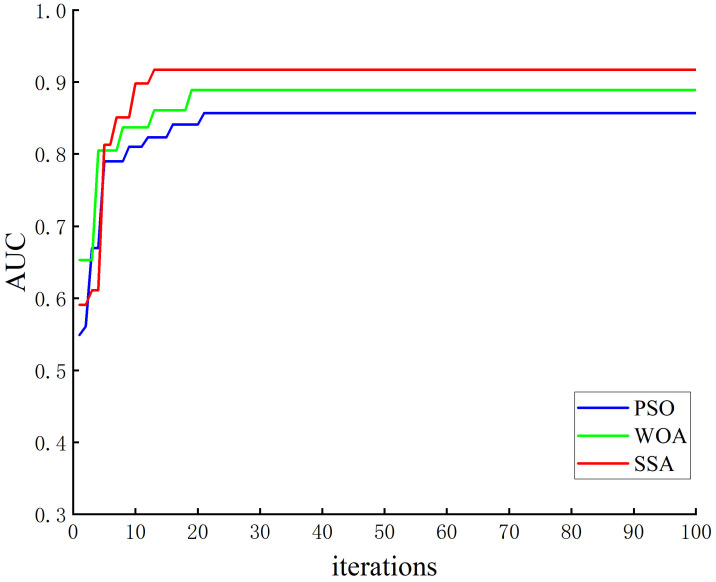
Fitness curve of each optimization algorithm.

As the selection of machine learning parameters is a non-linear optimization problem, traditional optimization algorithms have unavoidable drawbacks. The best combination of parameters cannot be obtained quickly, and they are even more complicated when used in reality. Therefore, swarm intelligence optimization algorithms were employed to select the parameters of the models, providing better solutions even in the face of difficult problems [42]. Common swarm intelligence optimization algorithms are PSO, WOA, and SSA, and have been widely used by researchers for machine learning [43,44,45], as well as having advantages in hyperparameter search for CatBoost, converge quickly, and improve CatBoost’s performance [46,47,48]. Therefore, in this study, PSO, WOA, and SSA were adopted to optimize the four hyperparameters of the CatBoost model, and the results were compared. The AUC of the validation set was taken as the fitness value to create the fitness function curve. Figure 9 shows the fitness curves of the corresponding optimization algorithms.

As seen in Figure 9, the SSA was the first to achieve the optimal result; the number of iterations to attain the optimal fitness was 13, and the optimal fitness value was 0.917. The optimal fitness value was 0.889 for the WOA, and the number of iterations to attain optimal fitness was 19. The final fitness value for PSO is the smallest at 0.857. The number of iterations to achieve optimal fitness was 21, which was the worst optimization. The best combination of parameters achieved by different optimization algorithms is shown in Table 10.

**Figure 10:**
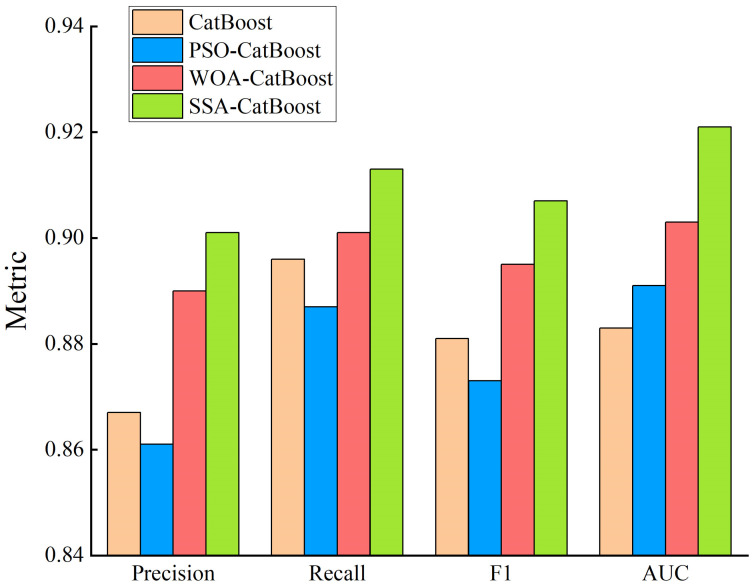
Evaluation comparison of different models.

CatBoost and CatBoost optimized via three algorithms were used to classify the ecological suitability of blueberries. A comparison of the assessment results is shown in Figure 10 and the training time comparison is shown in Table 11.

The figures demonstrate that the optimized CatBoost model performs better than the CatBoost model, except for the PSO-CatBoost model, in which the SSA-CatBoost model achieves the best performance in all aspects, with an AUC value of 0.921, and it has the best generalization ability. Regarding training time, the optimized CatBoost model takes less time to train than the unoptimized model, with the SSA-CatBoost model taking the fastest time, whose training time was 7 s.

In summary, compared with other optimization algorithms, SSA has the best optimization effect and the SSA-CatBoost model is more suitable for the ecological suitability classification of blueberries.

### 5.4. Feature Importance Analysis

**Figure 11:**
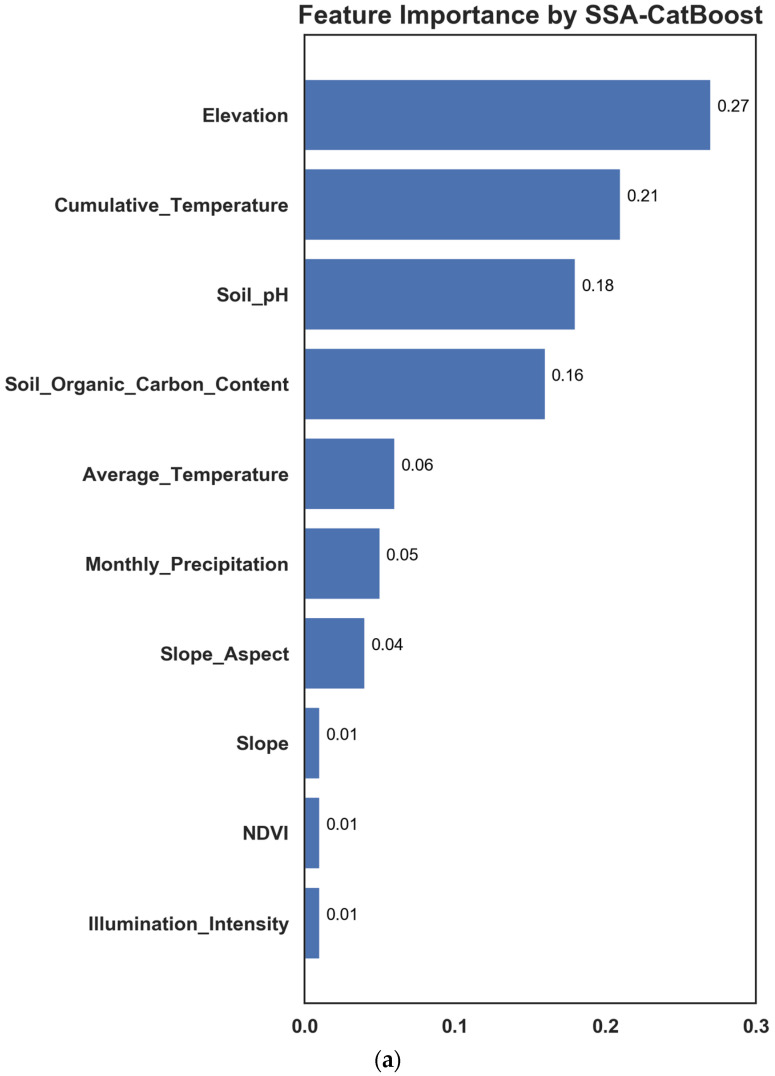

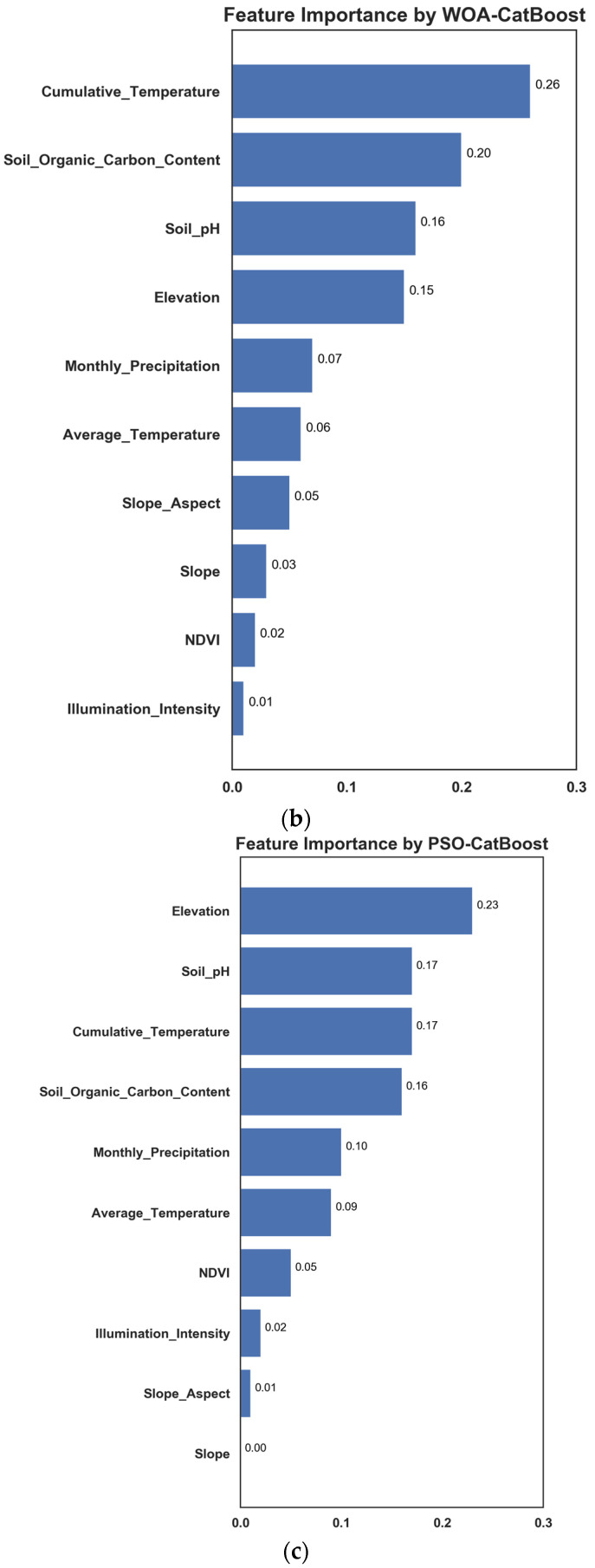
(**a**) Feature importance using SSA-CatBoost; (**b**) feature importance using WOA-CatBoost; (**c**) feature importance using PSO-CatBoost.

The feature importance of the SSA-CatBoost, WOA-CatBoost, and PSO-CatBoost was calculated, and the results are shown in Figure 11. It can be seen that the contribution of the elevation, cumulative temperature, soil organic carbon content, and soil pH obtained high scores in different models with mean values of 0.2167, 0.2133, 0.1733, and 0.17, respectively. The sum of the contributions accounted for 77.33% of the total sum of all the features, which indicated that these four features had a higher effect on the ecological suitability of blueberries and were significant factors affecting the growth of blueberries. Conversely, the slope, slope aspect, NDVI, and illumination intensity had low contributions with mean values of 0.01, 0.0334, 0.0267, 0.0133, respectively, with the sum of contributions accounting for 8.34% of the total sum of all the features.

### 5.5. Analysis of the Effect of Different Inputs on the Model

The performance of the classification model was strongly correlated with the sample feature data, and the effects of the different feature inputs on the performance of the classification model were discussed.

**Figure 12:**
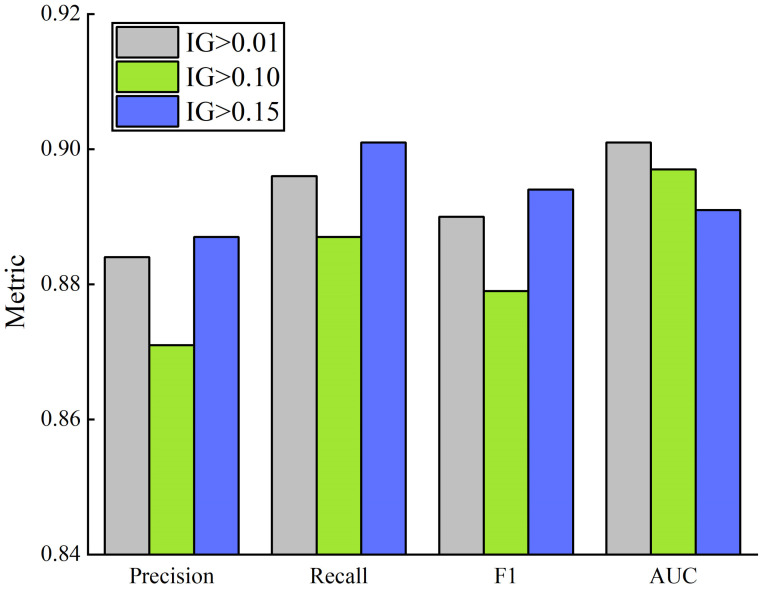
Comparison of models based on inputs with different IG indices.

The datasets were divided according to the different IG values. The table reveals that the IG values of the ten selected features are all greater than zero. Each feature affects the expressiveness of the blueberry ecological model. The sample data were then divided into three categories according to the different IG values of the features, IG > 0.01 (all features), IG > 0.1 (features: elevation, monthly precipitation, ≥10 °C cumulative temperature, illumination intensity, soil pH, soil organic carbon content) and IG > 0.15 (features: elevation, ≥10 °C cumulative temperature, soil pH, soil organic carbon content) which were tested using the SSA-CatBoost classification model and the model evaluations based on the different datasets are shown in Figure 12.

As can be seen from Figure 12, the model based on a dataset of IG > 0.01 had the highest AUC value, which was higher than IG > 0.1 and IG > 0.15, indicating that when each feature contributes to the model, the higher the input feature dimension, the more adequately trained the model is, the more robust the model is and the better generalization performance it has. Using the feature dataset of IG > 0.15 (four features: elevation, soil organic carbon content, soil pH, ≥10 °C cumulative temperature) as the model input, the model had comparatively better classification ability, exhibiting good performance on accuracy, recall, and so on. This suggests that the classification ability of this SSA-CatBoost model is closely related to the input of features with an extensive IG index.

**Figure 13:**
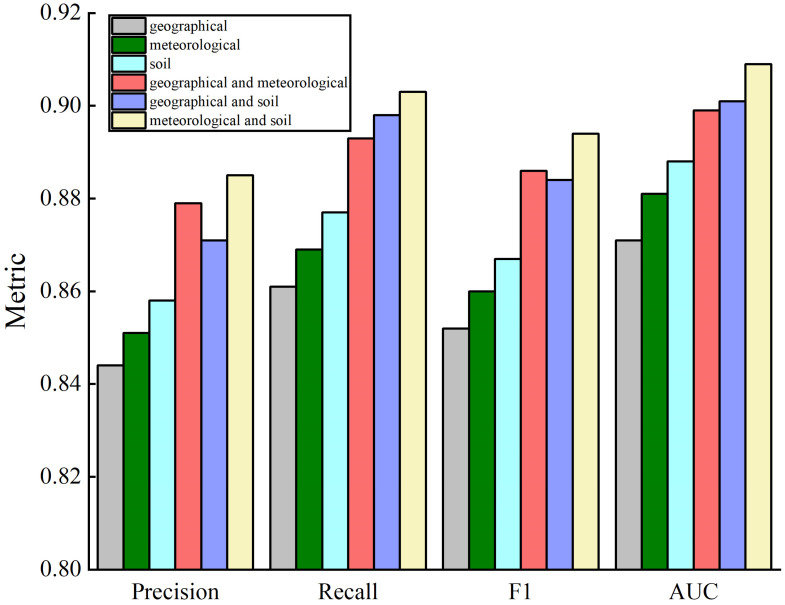
Comparison of models based on inputs from different environmental factor datasets.

The datasets were divided according to different environmental factors. The growth of blueberries is primarily governed by geography, meteorology, and soil. Therefore, the sample data were sorted into three other datasets: geographical (elevation, slope, slope aspect, NDVI), meteorological (monthly precipitation, average monthly temperature, ≥10 °C cumulative temperature, illumination intensity) and soil (Soil pH, soil organic carbon content), and single and multiple datasets were used as different data inputs, and tested using the SSA-CatBoost classification model. The model evaluation on the different data sets is shown in Figure 13.

As shown in Figure 13, comparing the performance of the model when only a single class of feature set was available as input, soil features had the most remarkable effects on the performance of the model, and the SSA-CatBoost model performed well in all aspects, indicating a high degree of influence of soil environmental factors on blueberry growth, followed by meteorological features and geographical features.

When datasets with multiple environmental features (geo-meteorological, geo-soil, or meteorological-soil) were used as model inputs, the model outperformed when datasets with a single feature were taken as the inputs. In particular, the model performed very well when the meteorological-soil feature was inputted into the model. The multi-type feature set provides more complete and representative information about the blueberry growing environment than the single-type feature set.

### 5.6. Mapping the Ecological Suitability of Blueberries

The machine learning model was employed to classify the ecological suitability of blueberries across the county of Majiang. The classification probability of each unit (30 m × 30 m) was saved as an ArcGIS attribute table and presented in a visual form, which would be more intuitive and practical for direct judgment based on the ecological suitability map of blueberries.

In the machine learning chosen for this paper, the classification results had a natural probability meaning, except for the SVM, where a probability value of zero meant unsuitable for blueberry growth and a probability value of one indicated suitable for blueberry growth, with increasing levels of ecological suitability for blueberry from zero to one. The SVM model cannot directly give the probability of classification results. However, the model prediction via five-fold cross-validation also fulfils the requirement that the results have a natural possibility meaning.

**Figure 14:**
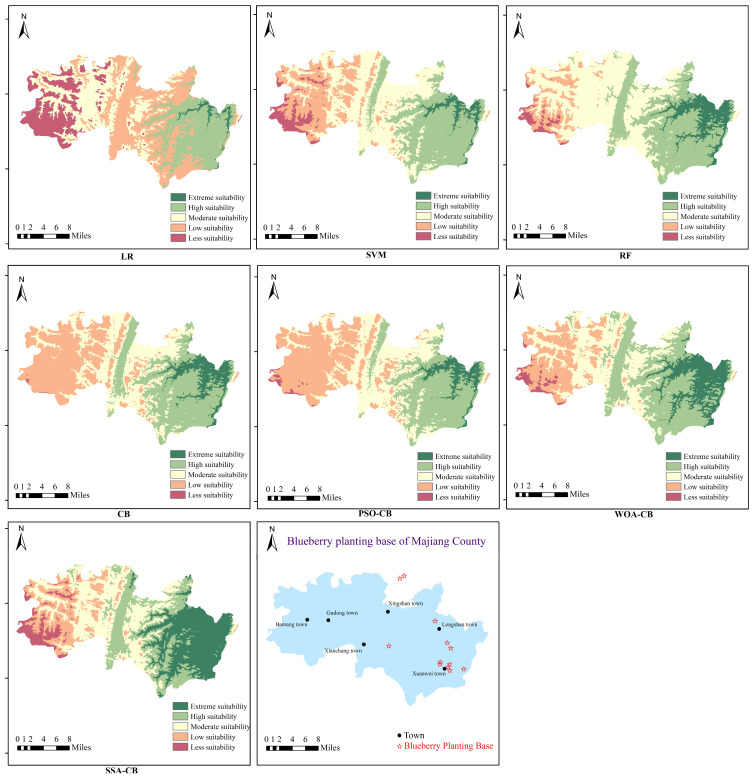
Ecological suitability evaluation map of blueberry in Majiang County with different models.

The ecological suitability of blueberries in Majiang County was classified into five classes: [0–0.20) less suitability, [0.2–0.40) low suitability, [0.40–0.60) moderate suitability, [0.60–0.80) high suitability, and [0.80–1] extreme suitability. The map of the ecological suitability evaluation of blueberries in Majiang County with different models is shown in Figure 14.

Most blueberry planting bases in Majiang County are located in the southeastern region. Market surveys have shown that the blueberries in this area have better quality and a higher yield. As shown in Figure 8, the suitability classification results of the integrated learning model were more compatible with the practical growing condition of blueberries than those of the single model. The classification results of the SSA-CatBoost model revealed that the extreme suitable and higher suitable areas for blueberry growth were mainly located in Xuanwei Town and Longshan Town in the southeastern part of the county, and the classification results matched the actual growing area the best compared with the classification results of other models. It can be seen that the classification model of blueberry ecological suitability constructed through the SSA-CatBoost model is effective and reliable and has significant reference value for the selection of blueberry planting areas.

## 6. Discussion

### 6.1. Main Findings

In this study, four machine learning (LR, SVM, RF, and CatBoost) were applied to build an ecological suitability classification model for blueberries. We gathered data on multiple sources of environmental features affecting the blueberry’s growth, balanced positive and negative samples of blueberries by the Borderline-SMOTE algorithm, and filtered features via VIF and IG. The processed data were then treated as input to the models for training. Ultimately, it was found that the performance ranking of the four machine learning models was CatBoost > RF > SVM > LR. Among the four machine learning models, CatBoost had the best performance metric among the four machine learning models, with an AUC of 0.897. In addition, we also conducted the hyperparametric search for CatBoost using SSA and found that the SSA-CatBoost model had a superior performance. Therefore, the SSA-CatBoost can be applied as the best model in the machine learning-based blueberry ecological suitability classification model.

### 6.2. Model Performance

Comparing the characteristics of the LR, SVM, RF, and CatBoost classification models, the ensemble learning classification models outperformed the single machine learning models. Among them, CatBoost outperformed all evaluation metrics. After optimizing the CatBoost hyperparameters with different optimization algorithms, the performance of the model was further improved while reducing the training time, and the AUC ranking of each model was SSA-CatBoost (0.921) > WOA-CatBoost (0.903) > PSO-CatBoost (0.891).

### 6.3. Main Features

In this study, machine learning technology was used to rank the importance of environmental features affecting blueberry growth, and a comprehensive analysis was made from the feature importance scores of each model. Elevation, ≥10 °C cumulative temperature, soil organic carbon content, and soil pH had high contribution values to the models, indicating that they had an increased influence on the ecological suitability of blueberries and were the most important factors affecting blueberry growth. On the contrary, slope, slope aspect, NDVI, and illumination intensity had lower contribution values to the model, indicating that they have less effect on the ecological suitability of blueberries. In Majiang County, blueberry cultivation bases are mainly located in the southeastern part of the county, where the altitude is low, the temperature is suitable all year round, and the soil pH is stable between 4.5 and 5.5, which is suitable for blueberry growth. Conversely, the northwestern part of the county has high altitudes and cold temperatures, which is not suitable for the development of blueberries.

### 6.4. Model Inputs

This study examined the variation in performance and the applicability of the model among different datasets taken as inputs. When datasets with different dimensions or datasets with various environmental features were adopted as model inputs, the performance and classification results of the model were significantly affected. For the model of blueberry ecological suitability proposed in this paper, the higher the feature dimension of the dataset, the better the model can be trained and the better the classification performance; when datasets with different environmental factors were used as model inputs, the influence of soil and meteorological factors on the classification results was more obvious, which indirectly indicates that soil and meteorological factors have a more substantial effect on the growth of blueberry. Therefore, the choice of the dataset is crucial to the performance of the model and the classification results. If sample data from other crops meet the requirements of this suitability classification method, the method can be extended to the suitability classification of other crops.

### 6.5. Model Advantages

CatBoost is the core of the model proposed in this paper, and as a representative of the Boosting family of algorithms, it has demonstrated its superiority in many cases. Qin et al. [49] built a prediction model for diabetes using LR, SVM, RF, XGBoost, and CatBoost, in which the AUC score of CatBoost was higher than the other models by 0.83. Zhang et al. [50] used machine learning to build a model for identifying depression in middle-aged and elderly people, and again, CatBoost performed better than LR, SVM, RF, and Back Propagation (BP). The AUC of CatBoost was higher than that of LR, SVM, RF, and the Artificial Neural Network (ANN) in building a landslide susceptibility prediction model by Wang et al. [51]. The model developed with CatBoost could better solve the gradient bias and prediction bias problems, thus reducing the occurrence of overfitting and improving the accuracy and generalization ability of the model. Based on the problem of unbalanced blueberry suitability sample data, this study proposed the Borderline-SMOTE algorithm to balance the positive and negative samples of blueberries to prevent the model from causing incorrect classification results under the condition of unbalanced data set classes.

## 7. Conclusions

Although machine learning technology has been increasingly used in smart agriculture, there is a lack of research on the suitability classification of crops in the case of multi-source environmental characteristics. In this study, machine learning techniques are applied to the ecological suitability classification of blueberries for the first time, and the experimental results demonstrate the effectiveness and applicability of the proposed method. We used 918 cases of sample data from the blueberry plantation in Majiang County, Guizhou Province, China, the Borderline-SMOTE algorithm to balance the number of positive and negative samples, followed by the use of the Variance Inflation Factor and information gain methods to screen out the characteristic factors affecting blueberry growth. The processed data were then fed into each machine learning model for training. After model training, the AUC was applied to evaluate the model; finally, the performance ranking of the four machine learning models was evaluated as CatBoost > RF > SVM > LR. In addition, using the optimization algorithm to optimize the parameters of CatBoost, we found that SSA-CatBoost performed the best, with an AUC of 0.921. Our comprehensive experimental results showed that the proposed model could better classify the ecological suitability of blueberries.

Notably, there are certain requirements on data quality when using machine learning to solve practical problems. In the future, our research will be more inclined to feature data, and we will collect more feature data that affect blueberry growth. The influence of different features on model performance and classification results will also be included in subsequent studies for further optimization of the model to obtain better model classification performance.

## Figures and Tables

**Table 1:** Data sources.

Data Type	Data Source	Years
Geographic data	Geospatial data (http://www.gscloud.cn, accessed on 3 May 2022)	2021
Meteorological data	Meteorological Bureau of Guizhou Province (http://gz.cma.gov.cn, accessed on 3 May 2022)	2018
Soil data	Soil Science Database (http://vdb3.soil.csdb.cn, accessed on 3 May 2022)	2018

**Table 2:** Partial sample data.

Number	Elevation (m)	Slope (°)	Slope Aspect	NDVI	Monthly Average Temperature (°C)	≥10 °C Cumulative Temperature (d·°C)	Illumination Intensity (Lux)	Soil pH	Soil Organic Carbon Content (g/kg)	Quality of Blueberries
1	756	27	North-east	3.7	20.7	3666	4913	4.8	5.1	High
2	1350	18	West	10.1	10.3	5431	11,254	6.8	1.7	Low
3	689	17	Flat	2.7	13.9	3875	7964	4.6	3.6	High
4	901	18	Flat	4.1	14.3	4233	6582	4.6	2.0	High
5	1465	25	North	11.0	11.7	2981	8974	6.9	3.8	Low
6	1227	15	North	6.1	10.1	5513	7512	5.7	3.6	Low
7	1228	9	Flat	2.9	11.9	4192	12,641	5.7	4.1	Low
8	1090	7	South-east	5.4	12.1	3112	3347	5.6	1.7	Low
9	717	11	East	7.3	21.4	3751	7994	4.3	3.9	High
10	689	26	Flat	3.4	11.2	2983	5971	4.7	5.8	High
11	1210	27	North	8.5	12.8	2469	11,342	5.2	5.1	Low
12	631	1	North-east	5.1	24.1	4891	6034	4.2	6.7	High

**Table 4:** Sample data table.

Number	Elevation	Slope	Slope Aspect	NDVI	Monthly Average Temperature	≥10 °C Cumulative Temperature	Illumination Intensity	Soil pH	Soil Organic Carbon Content	Ecological Suitability
1	2	3	3	2	4	2	4	3	3	1
2	5	2	8	4	3	3	6	4	1	0
3	1	1	1	1	3	2	5	3	2	1
4	3	1	1	2	3	3	4	3	1	1
5	6	2	2	4	2	1	5	4	2	0
6	5	2	2	3	3	4	5	4	2	0
7	5	1	1	1	2	1	6	4	3	0
8	4	2	5	2	3	2	3	4	1	0
9	2	2	4	3	4	2	5	2	2	1
10	1	1	1	1	3	1	4	3	3	1
11	4	4	2	3	2	1	6	3	3	0
12	2	2	3	2	4	3	4	2	4	1

**Table 5:** VIF and IG for the features of ecological suitability.

Number	Feature	VIF	IG
1	Elevation	1.948	0.266
2	Slope	1.620	0.032
3	Slope aspect	1.031	0.027
4	NDVI	1.718	0.076
5	Monthly precipitation	1.256	0.128
6	Monthly average temperature	1.134	0.093
7	≥10 °C cumulative temperature	1.537	0.193
8	Illumination intensity	1.371	0.136
9	Soil pH	2.109	0.198
10	Soil organic carbon content	1.441	0.271

**Table 6:** Comparison of sample data sets.

Data	Number of Samples	Positive Samples (Well-Suited)	Negative Samples (Non-Well-Suited)	Number of Features
Before processing	918	632	286	10
After processing	1106	632	474	10

**Table 7:** Main parameters of different models.

Model	Main Parameters
LR	*penality=*l2, *C=*2, *l1_ratio=*0.3, *max_iter=*100, *solver=*‘lbfgs’, *class_weight=*None*, random_state=*None
SVM	*C=*8, *gamma=*0.3, *kernel=*’rbf’, *probability=*False, *gamma=*’Scale*’, max_iter=*1000, *degree*=3
RF	*n_estimators=*80, *max_depth=*3, *min_samples_split=*2, *min_samples_leaf=*5, *mini_impurity_decrease=*0.4, *criterion=‘*gini*’*, *boostrap=*Ture, *oob_score=*False, *max_leaf_node*=None
CatBoost	*loss_function=*’Logloss’, *eval_metric=*’AUC’, *task_type=*’CPU’, *learning_rate=*0.1, *iterations=*10, *depth=*6, *l2_leaf_reg*=10, *boosting_type=*‘*Ordered*’, *random_seed=*‘*123*’,

**Table 8:** Experimental results of different models.

Model	Evaluation Index			
	Precision	Recall	F1-Score	AUC
LR	0.768	0.851	0.807	0.855
SVM	0.883	0.823	0.851	0.864
RF	0.853	0.843	0.862	0.875
CatBoost	0.891	0.874	0.882	0.897

**Table 9:** The training time of different models.

Model	Training Time/s
LR	17
SVM	39
RF	17
CatBoost	21

**Table 10:** Optimal parameters of CatBoost for different optimization algorithms.

Optimization Algorithm	Optimal Parameters for CatBoost
PSO	*loss_function=*’Logloss’, *eval_metric=*’AUC’, *task_type=*’CPU’, *boosting_type=*‘*Ordered*’, *random_seed=*‘123’, *learning_rate=*0.03, *iterations=*100, *depth=*6, *l2_leaf_reg=*20
WOA	*loss_function=*’Logloss’, *eval_metric=*’AUC’, *task_type=*’CPU’, *boosting_type=*‘*Ordered*’, *random_seed=*‘123’, *learning_rate=*0.2, *iterations=*60, *depth=*8, *l2_leaf_reg=*27
SSA	*loss_function=*’Logloss’, *eval_metric=*’AUC’, *task_type=*’CPU’, *boosting_type=*‘*Ordered*’, *random_seed=*‘123’, *learning_rate=*0.1, *iterations=*50, *depth=*8, *l2_leaf_reg=*18

**Table 11:** The training time of different optimized models.

Model	Training Time/s
CatBoost	21
PSO-CatBoost	18
WOA-CatBoost	11
SSA-CatBoost	7

## Data Availability

The authors confirm that the data supporting the findings of this study are available within the Supplementary Materials.

## Supplementary Materials

The following supporting information can be downloaded at: https://www.mdpi.com/article/10.3390/s23041811/s1, Supplementary Material: The ecological suitability data of Blueberry in Majiang County, Guizhou Province, China.
